# Outcomes after arthroscopic scapulothoracic bursectomy for the treatment of symptomatic snapping scapula syndrome

**DOI:** 10.1016/j.jseint.2022.08.002

**Published:** 2022-08-17

**Authors:** Neil Gambhir, Matthew G. Alben, Matthew T. Kim, Yaniv Pines, Mandeep S. Virk, Young W. Kwon

**Affiliations:** Division of Shoulder and Elbow Surgery, Department of Orthopaedic Surgery, NYU Langone Orthopedic Hospital -NYU Langone Health, New York, NY, USA

**Keywords:** Scapulothoracic bursitis, Snapping scapula, Arthroscopic bursectomy, Pain relief, PROMIS upper extremity, PROMIS pain intensity, PROMIS pain interference

## Abstract

**Background:**

The purpose of our study was to examine the clinical outcomes after arthroscopic scapulothoracic bursectomy for the treatment of scapulothoracic bursitis at a minimum of 2-year follow-up.

**Methods:**

Twenty patients who underwent arthroscopic scapulothoracic bursectomy for the treatment of symptomatic snapping scapula syndrome were identified from a single surgeon’s database. Patients were indicated for surgery if their symptoms persisted for more than 6 months and if they failed nonoperative treatment. Acquired data included patient demographics, shoulder range of motion, American Shoulder and Elbow Surgeon score, visual analog scale scores for pain, and the following Patient-Reported Outcomes Measurement Information System scores: Upper Extremity Computer Adaptive Test Version 2.0, pain intensity, and pain interference scores. Patient satisfaction and subjective shoulder value were also recorded out of 100. Fisher's test and unpaired *t* tests were performed for statistical analysis, and *P* values <.05 were considered significant.

**Results:**

A total of 20 patients (24 scapulae) were included in our study, with an average follow-up period of 44 (range: 27-91) months. The mean postoperative Patient-Reported Outcomes Measurement Information System scores for Upper Extremity Computer Adaptive Test Version 2.0, pain interference, and pain intensity were 44.2 ± 10.7, 50.9 ± 9.5, and 42.1 ± 9.5, respectively. The mean postoperative American Shoulder and Elbow Surgeon score was 79.0 ± 21.5, and the mean subjective shoulder value was 82.7 ± 12.9. Visual analog scale pain levels showed a significant decrease from 4.95 ± 2.26 preoperatively to 2.27 ± 2.7 (*P* < .05) postoperatively. There was no significant difference in shoulder range of motion after surgery. The mean patient satisfaction was 72.9, with 65% (13/20) of patients reporting satisfaction levels ≥ 80%. Two patients did not report the resolution of their symptoms and required revision surgery.

**Conclusion:**

Arthroscopic treatment of scapulothoracic bursitis is a safe, reliable technique that is effective in providing symptomatic relief with a low rate of recurrence, with most patients reporting a significant reduction in periscapular pain.

Scapulothoracic bursitis, also known as snapping scapula syndrome (SSS), is a painful condition characterized by grinding and crepitus within the periscapular region.^8^ SSS affects a wide demographic of patients, including both athletes and nonathletes alike. Exacerbated by overhead motions, and accompanied by pain in the neck and arm, scapulothoracic bursitis is a relatively uncommon condition whose treatment poses a challenge for both physicians and patients alike.

Symptomatic SSS can develop as a result of altered scapula positioning, presence of abnormal bony anatomy, or cervical nerve root irritation.^5^^,^^8^^,^^12^^,^^15^ Weakness of the periscapular muscles can result in scapular dyskinesis in which the scapula develops an anterior tilt, decreased upward rotation, and increased internal rotation.^2^^,^^16^ This altered positioning results in the scapula pivoting along its medial border rather than sliding laterally. The presence of an osteochondroma, rib deformity, or tubercle of Luschka can also lead to disruption of the natural gliding motion of the scapula and bursal irritation.^3^, ^4^, ^5^^,^^8^^,^^13^ Regardless of the cause, this nonphysiological motion results in an increased frictional force on the scapular bursae and subsequent development of painful bursal inflammation.

The mainstay treatment of SSS traditionally consists of conservative measures, including physical therapy, rest, and corticosteroid injections. When nonsurgical modalities fail, surgical intervention is often indicated. Traditionally, SSS has been managed with open, invasive surgeries in the form of superomedial angle resection, partial scapulectomy, and open bursectomy.^8^^,^^9^ In recent years, arthroscopic scapulothoracic bursectomy (aSTBy) has been used as a less invasive means of treatment with promising outcomes.^2^^,^^10^^,^^11^ The literature surrounding aSTBy, however, is relatively sparse, consisting of relatively small cohorts of patients. Therefore, the purpose of our study is to expand upon available information by reporting the functional outcomes, change in pain, and patient satisfaction of 20 patients who underwent aSTBy for the treatment of SSS with a minimum follow-up of 2 years to assess the efficacy of this surgical modality. We hypothesize that aSTBy can result in significant clinical improvements in this rare group of patients.

## Methods and materials

### Patient selection and data acquisition

Institutional review board approval was granted for this study. Twenty patients (8 males and 12 females) who underwent aSTBy for the treatment of nontraumatic SSS from 2012 to 2019 were identified from the senior author’s database and consented for their inclusion in our study. Patients were indicated for aSTBy for persistent pain lasting for more than 6 months after failing nonoperative treatment consisting of a supervised physical therapy program and a corticosteroid injection. All patients who consented were aged ≥18 years and native English speakers. Data were acquired during office visits and video visits and through manual chart review via Epic (Epic Systems Corporation, Verona, WI, USA). All patient data were stored within our institutional REDCap database (Vanderbilt University, Nashville, TN, USA).

Preoperative metrics acquired from patients included shoulder range of motion (ROM) and visual analog scale (VAS) pain scores. Metrics acquired at a minimum of 2 years after surgery included Patient-Reported Outcomes Measurement System (PROMIS) Upper Extremity (UE) Computer Adaptive Test (CAT) Version 2.0 scores, PROMIS Pain intensity scores, PROMIS Pain interference scores, American Shoulder and Elbow Surgeons (ASES) scores, subjective shoulder values (SSVs), shoulder ROM, and VAS pain scores. Specifically, SSV scores were used to gauge patients’ postoperative self-reported (subjective) shoulder function from a scale of 0-100 compared with their contralateral shoulder or what they consider should be a normal healthy shoulder if their contralateral shoulder is also affected. A value of 100 corresponded to shoulder function similar to the contralateral shoulder. Patient satisfaction was also acquired using a sliding visual scale and was phrased in our survey as “On a scale of 0-100, how satisfied are you with your surgical outcome.”

### Surgical technique arthroscopic scapulothoracic bursectomy

**Figure 1:**
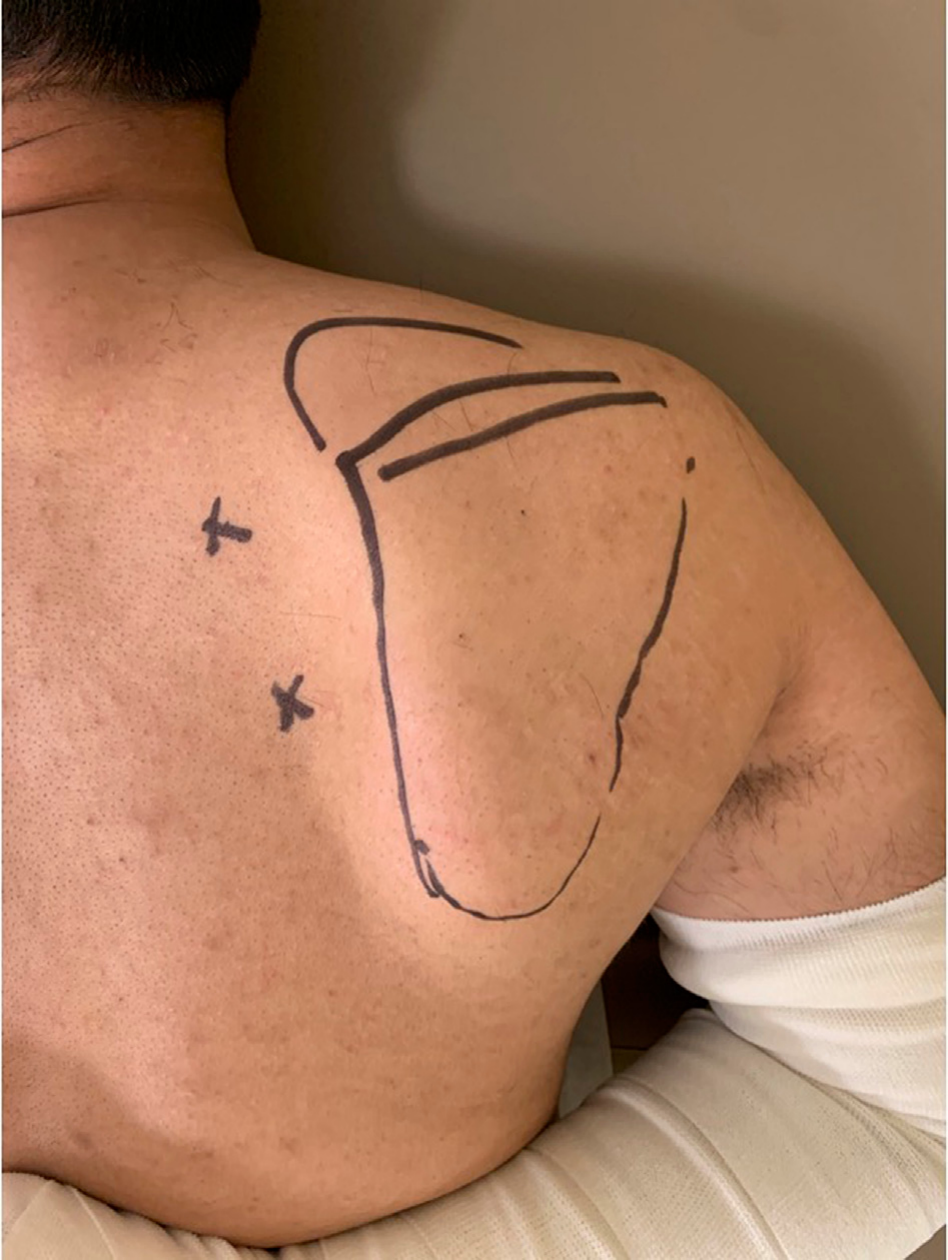
Arthroscopic portal sites (X) 3 cm medial to scapular border.

All patients underwent arthroscopic surgery in the prone position under general anesthesia. After the operative scapula and arm were prepped and draped, the arm was internally rotated and placed behind the back to move the medial border of the scapula away from the thoracic cavity. Two portals were established for arthroscopy. The first portal was 3 cm medial to the medial border of the scapular spine, whereas the second portal was located 3 cm medial to the midpoint between the scapular spine and inferior pole of the scapula (Fig. 1) to protect the neurovascular structures.

**Figure 2:**
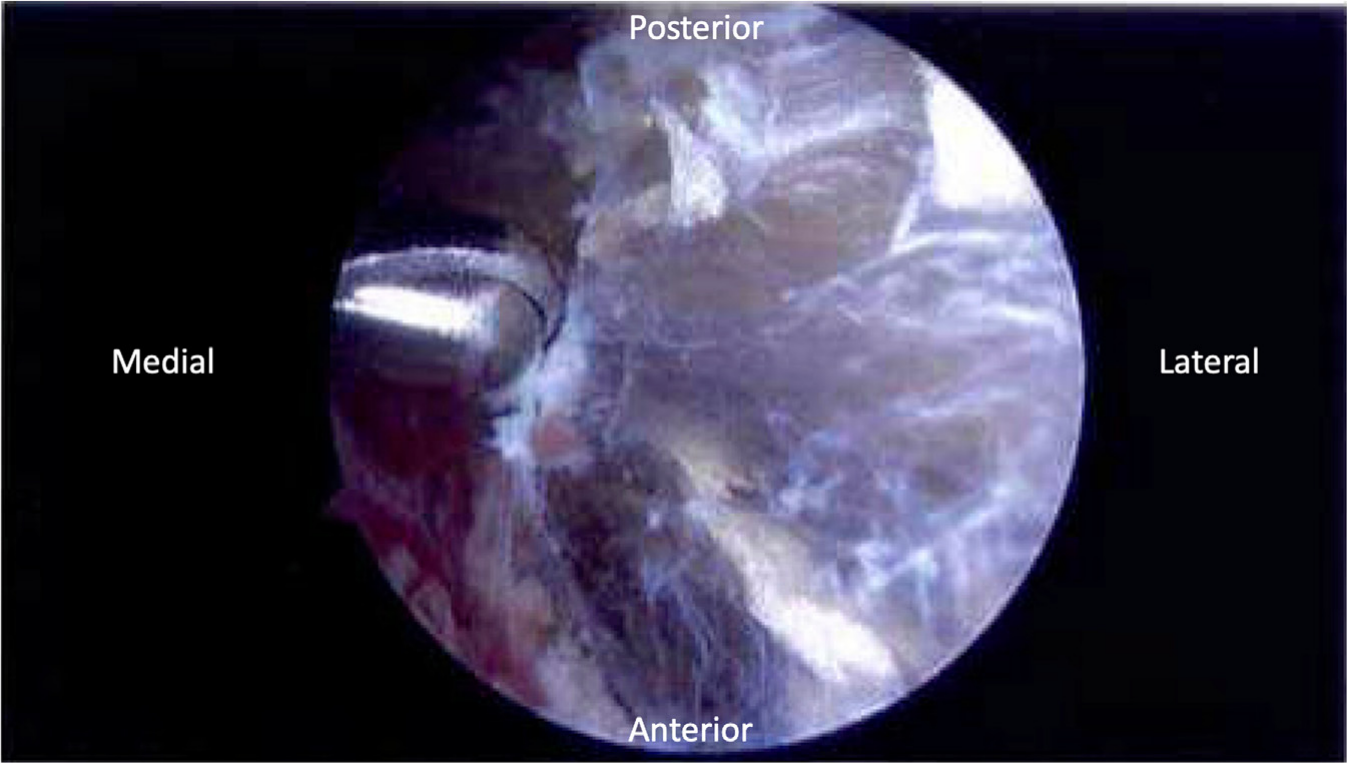
Arthroscopic view of scapulothoracic bursa.

**Figure 3:**
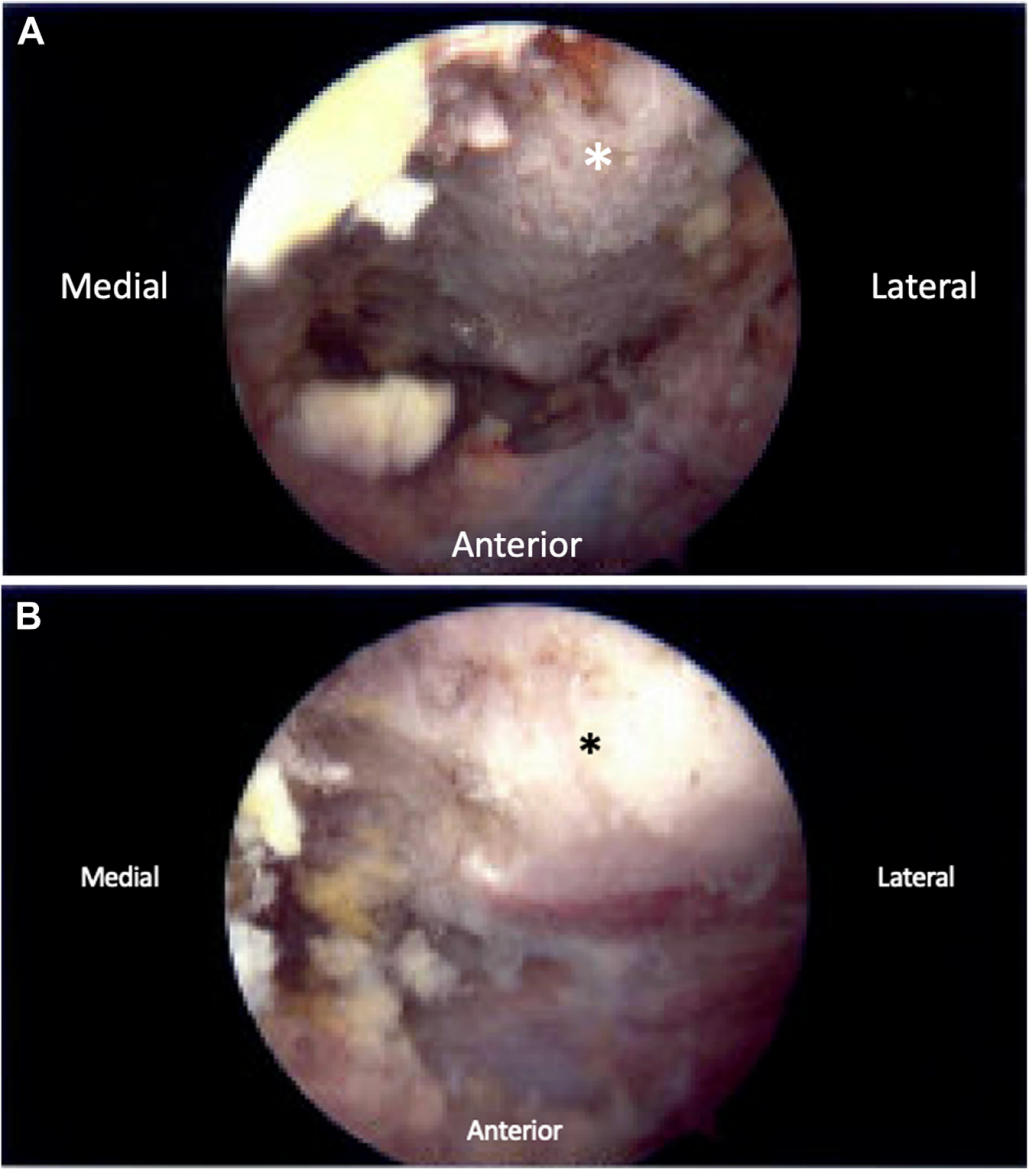
Arthroscopic image of anterior surface of the superior medial angle of the scapula (*∗*) before (**A**) and after (**B**) arthroscopic resection.

Using these portals interchangeably as viewing and working portals, the anteromedial aspect of the scapula was examined. The anterior surface of the scapula at the level of the scapular spine was explored while carefully protecting the nearby soft tissue structures. Scapulothoracic bursal tissue was débrided in its entirety with an arthroscopic shaver and arthroscopic radiofrequency ablator (Fig. 2). After bursectomy, the superomedial angle of the scapula was exposed, and bony prominences or the presence of an anterior tilt was resected with an arthroscopic burr to allow for its undersurface to be coplanar with the adjacent scapula (Fig. 3, A and B).

Following surgery, the operative arm was placed in a sling for 4 weeks. Patients were instructed to remove the sling for hygiene-related and waist-level activities. Patients began a supervised therapy program after 2 weeks beginning with exercises for active and passive motion for a total of 4 weeks. Gentle resistive exercises of the shoulder girdle were then initiated at 6 weeks after surgery. Patients were cleared to begin general strength training at 3 months from their operation.

### Statistical analyses

Statistical analysis was performed in SPSS Statistics 26.0 (IBM Corp., New York, NY, USA). Descriptive statistics were calculated for both categorical and continuous variables. Quantitative data were expressed as mean and standard deviation (mean ± SD), and qualitative data were expressed as percentages. To optimize the comparison of preoperative and postoperative internal rotation ROM, a numerical value ranging from 0 to 7 was assigned as follows: T7 (0), T8 (1), T9 (2), T10 (3), T11 (4), T12 (5), L1 (6), and L2 (7) as previously described.^18^ An unpaired *t* test was conducted to compare continuous data, and Fisher's exact test was conducted to compare categorical data. A *P* value < .05 was considered statistically significant.

## Results

Twenty patients (24 scapulae) with a mean age of 40 (range 22-74) years at the time of surgery were included in our study. Baseline patient demographics and characteristics can be seen in Table I. No significant differences between male and female patients with regard to body mass index or smoking status were found. Preoperative imaging in the form of radiographs was negative in all patients for the presence of bony abnormalities, such as osteochondroma, rib deformity, or tubercle of Luschka. In addition, no patient had evidence of a contributory cervical radiculopathy.

**Table I:** Patient demographics.

Variables examined	Male	Female	*P* value
Count (N)	8	12	-
Age	39.0 ± 11.3	43.7 ± 16.4	.494
BMI	25.5 ± 4.0	26.1 ± 5.1	.773
Smoking history	3/8 (37.5%)	4/12 (33.3%)	.848

*BMI*, body mass index.

Patient-reported outcomes were obtained at 66.2 (range 27-98) months after surgery. The mean and individual patient scores can be seen in Table II. When stratified by gender, no significant differences between PROMIS or ASES scores were noted. There was a statistically significant difference in VAS pain scores from 4.95 ± 2.26 preoperatively to 2.27 ± 2.7 postoperatively (*P* = .012). SSV scores were acquired for 16/24 scapulae, with a mean score of 82.7 ± 12.9.

**Table II:** Individual patient PROMIS and laterality of scapula operated on.

Patient	Laterality	PROMIS UE	PROMIS interference	PROMIS intensity	ASES	SSV (%)
1	R	61	38.7	30.7	100.0	98
2∗	L	34.6	56	42.6	68.3	-
3	R	34.6	61.5	53.7	56.7	80
4	L	37.1	56.6	43.6	83.3	100
5	R	29.2	61.5	51.3	83.3	-
6	R	54.5	38.7	35.8	86.7	-
7	R	32.5	71.6	58.6	45.0	50
8	R	61	38.7	30.7	98.3	85
9	L	40.5	50.1	42.6	73.33	78
10	L	40.1	50.1	51.9	78.3	75
11	R	31.2	65.3	58.6	50.0	-
12	R	40.5	47.4	42.6	90.0	90
	L	35.5	61.5	53.7	55.0	-
13	R	61	38.7	30.7	100.0	-
	L	40.5	46.6	35.8	93.3	-
14∗	R	34.3	52.6	40.5	56.7	70
	R	50.6	46.6	35.8	88.3	-
15	R	45.3	46.6	35.8	88.3	75
	L	55.9	38.7	30.7	100.0	90
16	R	61	38.7	30.7	100.0	97
17	R	46.7	52.6	51.3	68.3	80
18	R	34.6	56	42.6	21.7	83
19	R	42.4	46.6	30.7	98.3	97
20	L	55.9	55.8	49.9	100	75
Mean ± STD		44.2 ± 10.7	50.9 ± 9.5	42.1 ± 9.5	79.0 ± 21.5	82.7± 12.9

*PROMIS*, Patient-Reported Outcome Measurement Information System; *PROMIS UE*, PROMIS Upper Extremity Computer Adaptive Test Version 2.0; *ASES*, American Shoulder and Elbow Surgeons score; *SSV*, Subjective Shoulder Value; *STD*, standard deviation.

∗ Patient needing revision surgery.

**Figure 4:**
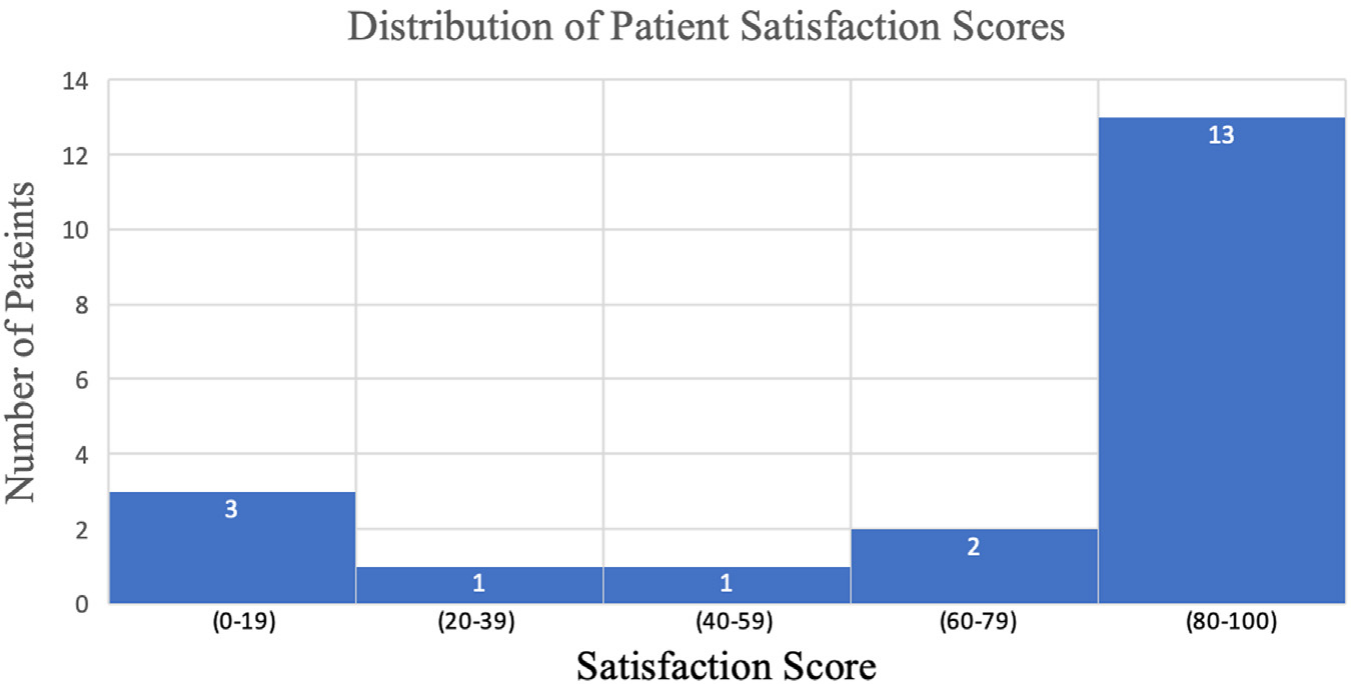
Histogram of patient satisfaction.

In addition, there were no significant differences between preoperative and postoperative shoulder ROM in forward elevation (175.5° ± 10.6° preoperative vs. 175.0° ± 12.3° postoperative; *P* = .90), external rotation (57.3° ± 9.7° preoperative vs. 59.1° ± 11.1° postoperative; *P* = .57), or internal rotation (2.8 ± 1.9 preoperative vs. 3.4 ± 1.7 postoperative; *P* = .33). The average patient satisfaction level was 72.9 (range 0-100), with 65% (13/20) of patients reporting satisfaction levels ≥80/100. The distribution of patient satisfaction levels can be seen in Fig. 4.

Two patients (10%) required revision surgery for continued pain, one of which reported resolution of symptoms after their revision arthroscopic surgery. Of the 8 patients who played sports, 4 (50%) stated that they were able to return to their sport (baseball, competitive dancing, weightlifting, swimming) at a level similar to or better than their baseline. Patients who were unable to return to sport reported pain and fear of injury as the limiting factors. There were no complications following aSTBY such as hematoma formation or the development of a neuropathy and no pneumothorax.

## Discussion

The findings of this study indicate that aSTBy is a safe, reliable technique that is effective in providing symptomatic relief with a low rate of recurrence. Although there was a meaningful improvement in pain scores, complete symptomatic resolution did not occur in several patients resulting in some residual upper extremity disability.

Prior studies involving aSTBy have not used PROMIS scores as outcome metrics. PROMIS instruments have been increasingly used for assessing outcomes in orthopedic surgery and provide an advantage over legacy scores in that they can be updated to improve scoring, adjust calibration, expand item banks, and improve patient responsiveness. PROMIS is measured on the T-score metric, with the mean of 50 and SD of 10 set to equal the mean of the US general population and scores ranging from approximately 15 to 60. A higher score indicates more of that domain being measured; a higher UE CAT indicates higher upper extremity physical functional level.^17^

The lower bound of our cohort’s PROMIS pain intensity score of 42.1 ± 9.5 fell one SD below this following aSTBy, indicating that aSTBy patients reportedly experienced pain relief that may be lower than the general US populace. Decreased levels of pain were also evident by a significant decrease in postoperative mean VAS pain scores. Of note, PROMIS pain interference scores of 50.9 ± 9.5 were nearly similar to the reference mean’s, which may indicate that our cohorts experience no difference in pain interference to the general US populace.

In regard to upper extremity function, PROMIS UE CAT, ASES, and SSV scores expressed variability among patients, with some reporting excellent function and others marked limitation after their operation (Table II). This can potentially be attributed to the fact that several patients in our series had concomitant shoulder pathologies (n = 11) and one patient having a history of a mixed connective tissue disorder. However, our mean ASES score of 79.0 ± 21.5 was consistent with previously published studies.^11^^,^^14^

Millet et al evaluated 21 patients (23 scapulae) with a mean age of 33 years, 2 years after receiving aSTBy.^14^ A significant improvement in the median ASES score was observed with an increase from 52 to 73 (*P* < .001). Similarly, a large retrospective review by Menge et al of 74 scapulae revealed a similar increase in ASES scores from 52.6 to 75.8 (*P* < .001).^11^ Interestingly, Menge’s study examined the psychosocial aspect of SSS via incorporating Short Form 12 health survey mental component scores into postoperative metrics. An increase was observed from 45.0 to 49.6 (*P* = .023), and a direct correlation was revealed between low preoperative psychological scores and lower postoperative outcome scores.^11^

Significant residual pain was still a factor in 4 patients in our cohort, with 2 requiring revisions. It is plausible that some bursae were not fully excised due to the inherent limitations of arthroscopic procedures. The presence of superomedial adventitious bursae in the serratus and fibrotic tissue have both been implicated as sources of continued pain after bursectomy.^6^^,^^14^ If identified on advanced imaging, or suspected, open procedures should be considered, and patients should be counseled accordingly.

As aforementioned, no patient in our series had any radiological evidence of a discrete causative bony abnormality, such as osteochondroma, rib deformity, or tubercle of Luschka. Therefore, the most likely cause of altered scapular motion in our patients was that of periscapular muscle weakness. In patients without these bony abnormalities, scapular and shoulder girdle physical rehabilitation programs have shown success in the treatment of SSS. The focus of these programs largely consists of postural education and muscular strengthening.^3^^,^^7^ Recently, extracorporeal shock wave therapy has shown promise in pain relief and may serve as an adjunct in facilitating nonoperative treatment.^1^

Our study was limited by its sample size, presence of concomitant shoulder pathologies, and lack of preoperative PROMS, although we believe a remarkable increase would have been observed. Furthermore, reference cohorts consisting of patients who have undergone open surgery and nonoperative treatment would have also been beneficial. At this point, no study has yet compared outcomes following open and arthroscopic surgeries. Future work is required to truly evaluate the optimal treatment modality for SSS.

## Conclusion

Arthroscopic treatment of scapulothoracic bursitis is a safe, reliable technique that is effective in providing symptomatic relief with a low rate of recurrence, with most patients reporting a significant reduction in periscapular pain.

## Disclaimers

Funding: No grants, funding, or technical support were received by any of the authors for this project.

Conflicts of interest: The authors, their immediate families, and any research foundation with which they are affiliated have not received any financial payments or other benefits from any commercial entity related to the subject of this article.
